# The trajectory of osteoblast progenitor cells in patients with type 2 diabetes and the predictive model for their osteogenic differentiation ability

**DOI:** 10.1038/s41598-023-29677-8

**Published:** 2023-02-09

**Authors:** Mattabhorn Phimphilai, Peraphan Pothacharoen, Nipon Chattipakorn, Prachya Kongtawelert

**Affiliations:** 1 Division of Endocrinology, Department of Internal Medicine, Faculty of Medicine, Chiang Mai University, Chiang Mai, 50200 Thailand; 2 Thailand Excellence Center for Tissue Engineering and Stem Cells, Department of Biochemistry, Faculty of Medicine, Chiang Mai University, Chiang Mai, 50200 Thailand; 3 Cardiac Electrophysiology Research and Training Center, Faculty of Medicine, Chiang Mai University, Chiang Mai, 50200 Thailand; 4 Department of Physiology, Faculty of Medicine, Chiang Mai University, Chiang Mai, 50200 Thailand

**Keywords:** Stem cells, Diseases, Endocrinology

## Abstract

The fate of osteoprogenitor cells along with the progression of type 2 diabetes (T2DM) and factors determining the fate of those cells remains to be elucidated. This cross-sectional study included 18 normoglycemic, 27 prediabetic, and 73 T2DM to determine osteogenic differentiation across the continuum of dysglycemia and to construct a model to predict the fate of osteoprogenitor cells. This study demonstrated a preserved osteogenic differentiation ability of peripheral blood-derived mononuclear cells (PBMC) isolated from normoglycemic and prediabetic but a progressive decline in their osteogenic differentiation during the progression of T2DM. The rate of osteogenic differentiation rapidly declined by 4–7% annually during the first 10 years of diabetes and then slowed down. A predictive model composed of three independent risk factors, including age, duration of diabetes, and glomerular filtration rate, demonstrated an AuROC of 0.834. With a proposed cut-off of 21.25, this model had 72.0% sensitivity, 87.5% specificity, and 78.9% accuracy in predicting the fate of osteoprogenitor cells. In conclusion, this study provided a perspective on the osteogenic differentiation ability of the osteoprogenitor cells across a continuum of dysglycemia and a predictive model with good diagnostic performance for the prediction of the fate of osteoprogenitor cells in patients with T2DM.

## Introduction

Type 2 diabetes (T2DM) is a highly concerning disease worldwide because of its rapidly increasing prevalence and its impact on morbidity and mortality. T2DM has insulin resistance as a central pathophysiology. This insulin resistance usually occurs years before the diagnosis of diabetes, leading to a dysglycemic continuum ranging from modest hyperglycemia in prediabetes to persistent overt hyperglycemia in T2DM. This chronic hyperglycemic state contributes to multiple devastating complications, including atherosclerotic cardiovascular disease, retinopathy, nephropathy, neuropathy, and fragility fractures.

T2DM is well known to be associated with impaired bone quality and adverse skeletal outcomes, including decreases in bone turnover^1,2^, changes in bone microarchitecture^3^, and increases in the risk of fragility fractures, even though there is preservation of bone mineral density^4–6^. In addition, the risk of fragility fractures increases with poorer glycemic control^7–9^ and a longer duration of diabetes^10^ in T2DM. In association with a milder degree of hyperglycemia, individuals with prediabetes also consistently showed a state of low bone turnover^11,12^, but data pertinent to fragility fractures varied^10,13–15^. Several studies showed an increased risk of hip fractures in a prediabetic population^10,14^, while other studies demonstrated that hip fractures did not increase in a population with prediabetes^13,15^. Data relating to prediabetes has been found to be inconsistent regarding the increasing risk of fragility fractures, but there is an increased risk of fragility fractures in diabetes with poorer glycemic control and a longer duration of diabetes. Therefore, the higher degree and the longer period of hyperglycemic exposure could have an impact on fragility fractures.

The state of low bone turnover, characterized by a decrease in markers of bone formation and bone resorption, has been demonstrated in both prediabetes^11,12^ and diabetes^1,2^. In addition, the low bone turnover state with defects in bone formation has been demonstrated by a histomorphometric study in diabetes^16^. That state of low bone turnover with a defect in bone formation was found to be partly linked to an alteration in osteoblast development and function. Multiple preclinical studies demonstrated defects in osteoblast proliferation, differentiation, and survival in individuals with prediabetes^17,18^ and diabetes^19,20^. In humans, several studies illustrated defects in osteoblast proliferation and differentiation in diabetic individuals^21–23^. Multiple factors were shown in vitro to influence the deterioration of osteoblast development and survival in diabetes, including the direct effect of hyperglycemia per se^24,25^ and indirect effects of hyperglycemia from accelerated accumulation of advanced glycation end products (AGEs)^26–31^ and an enhanced inflammatory state^19,25,31,32^. However, those factors remain to be elucidated for their impacts on osteoblast development and survival in patients with type 2 diabetes.

Multiple previous studies demonstrated defects in differentiation toward osteoblasts of osteoprogenitor cells; however, each of those studies focused only on a specific group of the type 2 diabetic population^2,21–23^. Therefore, the fate of osteoprogenitor cells along with the course of diabetic progression and factors determining the fate of those cells, remains in need of characterization. This study aimed to determine osteogenic differentiation ability across the continuum of dysglycemic states in T2DM as well as construct a predictive model to predict the fate of osteoprogenitor cells in patients with T2DM.

Peripheral blood mononuclear cells (PBMC) can serve as a source of osteoprogenitor cells^33–35^. Valenti et al.^33^ showed the expression of multiple osteoblast-specific genes during differentiation of the PBMC, including *COL1A1* and *RUNX2*, and also the production of osteocalcin, the protein encoded by *BGALP*. Our previous studies also demonstrated the osteogenic differentiation of the PBMC-isolated from both non-diabetic and diabetic individuals^22,23,36^. To obtain the necessary stem cells in the least invasive measure, we used the PBMC-isolated from participants as the source of osteoprogenitor cells in this study.

## Results

### Demographic data, clinical characters, and biochemical parameters of study participants

The study enrolled 118 participants, including 18 with normoglycemia, 27 with prediabetes, and 73 with T2DM. In all three groups, age, gender, body mass index (BMI), systolic blood pressure (SBP), diastolic blood pressure (DBP), eGFR, and 10-year fracture risk as determined by FRAX^®^^37^ were comparable (Table 1). When the three groups were compared, serum triglycerides tended to be higher in the diabetic group while serum HDL-C tended to be lower in the diabetic group (Table 1), corresponding to atherogenic dyslipidemia established in patients with insulin resistance. Serum LDL-C in the T2DM group was the lowest, which corresponded to the highest rate of statins use in the T2DM group (Table 1). With comparable levels of blood pressure among the three groups, the usage rate of angiotensin-converting enzyme inhibitors (ACEI) or angiotensin II receptor blockers (ARB) in the diabetic population was the highest, while the usage rates of dihydropyridine calcium channel blockers (DHP-CCB) and thiazide-like diuretics were comparable in those three groups, suggesting the highest degree of hypertension in the T2DM group.

**Table 1:** Clinical characteristics of the study participants.

Parameter	Normoglycemia (n = 18)	Prediabetes (n = 27)	*p*-value^&^	T2DM (n = 73)	*p*-value^&&^
Age (years)	58.6 ± 9.0	61.6 ± 7.5	0.239	60.0 ± 7.5	0.441
Gender (% female)	11 (61.1)	20 (74.1)	0.357	45 (59.2)	0.385
BMI (kg/m^2^)	24.3 ± 2.6	24.6 ± 3.8	0.812	25.9 ± 4.1	0.160
SBP (mmHg)	126.8 ± 14.1	132.9 ± 12.4	0.131	131.2 ± 14.7	0.350
DBP (mmHg)	80.9 ± 9.4	76.4 ± 10.1	0.140	75.4 ± 9.6	0.102
IFG duration (years)	–	3.2 (2.2–4.3)	–	–	–
DM duration (years)	–	–	–	5.7 (4.7–6.9)	–
Microvascular complications (%)	–	–	–	40 (52.6)	–
Macrovascular complication (%)	–	–	–	10 (13.2)	–
FPG (mg/dL)	89.9 (86.7–93.2)	102.1 (97.9–106.4)	< 0.001	135.5 (126.9–144.6)	< 0.001
HbA1c (%)	–	5.9 ± 0.5	–	7.4 ± 1.4	< 0.001
eGFR (ml/min)	81.7 ± 18.1	87.8 ± 14.8	0.230	80.2 ± 17.7	0.151
Triglycerides (mg/dL)	80.7 (67.0–101.4)	90.4 (77.6–108.1)	0.368	108.9 (99.6–120.1)	0.006
LDL-C* (mg/dL)	113.9 (98.7–131.5)	97.4 (87.1–108.9)	0.078	87.6 (80.5–95.3)	0.012
HDL-C# (mg/dL)	67.1 ± 27.0	60.0 ± 14.8	0.334	52.6 ± 13.6	0.002
Anti-hyperglycemic agents [n (%use)]					
Metformin	0	1 (3.7)	1.000	69 (94.5)	< 0.001
Sulfonylureas	0	0	–	18 (24.7)	0.001
Insulin	0	0	–	7 (9.6)	0.171
Others	0	0	–	7 (9.6)	0.171
Drugs [n (% use)]					
ACEI or ARB**	5 (27.8)	12 (46.2)	0.218	53 (72.6)	0.001
DHP-CCB##	11 (61.1)	9 (34.6)	0.083	41 (56.2)	0.119
Thiazide-like diuretics	3 (16.7)	6 (23.1)	0.604	20 (27.4)	0.624
Statins	10 (55.6)	15 (55.6)	1.000	63 (86.3)	0.001
Fibrate	2 (11.1)	3 (11.1)	1.000	4 (5.5)	0.414
Calcium and vitamin D	3 (16.7)	4 (14.8)	0.874	5 (6.8)	0.180
FRAX: 10-year risk of hip fractures (FRAX-H) (%)	0.4 (0.2–0.7)	0.7 (0.4–1.2)	0.140	0.4 (0.3–0.5)	0.143
FRAX:10-year risk of osteoporotic fractures (FRAX-O) (%)	2.3 (1.9–3.0)	3.0 (2.4–4.0)	0.141	2.4 (2.1–2.6)	0.128
Pentosidine (ng/mL)	3.6 (2.8–4.6)	2.5 (2.0–3.1)	0.029	3.4 (2.9–4.1)	0.095
Soluble RAGE (sRAGE) (pg/mL)	531.7 (414.7–681.6)	459.9 (369.8–572.0)	0.375	460.3 (415.4–510.1)	0.498
sRAGE-Pentosidine ratio (pg/ng)	146.2 (102.3–208.9)	182.2 (132.8–250.0)	0.352	134.3 (110.5–163.2)	0.252
Interleukin-1β (pg/mL)	0.5 (0.3–0.8)	0.5 (0.3–0.7)	0.844	0.4 (0.3–0.5)	0.156
Tumor necrosis factor-α (pg/mL)	0.7 (− 0.4 to 2.2)	2.2 (1.3–3.3)	0.058	2.1 (1.6–2.6)	0.036

^&^Comparison between groups with normoglycemia and prediabetes.

^&&^ Comparison among groups with normoglycemia, prediabetes and T2DM.

*LDL-C: low-density lipoprotein cholesterol; #HDL-C: high-density lipoprotein cholesterol;

**ACEI: angiotensin-converting enzyme inhibitors; **ARB: angiotensin II receptor blockers; ^##^DHP-CCB: dihydropyridine calcium channel blockers.

Participants in the prediabetic group were diagnosed with IFG for 3.2 (95% CI 2.2–4.3) years. FPG and HbA1c in the prediabetic group were 102.1 (95% CI 97.9–106.4) mg/dL and 5.9 ± 0.5%, respectively. Patients in the T2DM group had a diagnosis of diabetes for 5.7 (95% CI 4.7–6.9) years. FPG and HbA1c in the T2DM group were 135.5 (95% CI 126.9–144.6) mg/dL and 7.4 ± 1.4%, respectively. Forty (52.6%) and ten (13.2%) patients with T2DM had chronic microvascular complications and macrovascular complications, respectively (Table 1). FPG levels were significantly different among the normoglycemic, prediabetic and T2DM groups [89.9 (95% CI 86.7–93.2) mg/dL vs 102.1 (95% CI 97.9–106.4) mg/dL vs 135.5 (95% CI 126.9–144.6) mg/dL, *p* < 0.001] (Table 2). The HbA1c level was also significantly higher in the T2DM group than in prediabetic patients (7.4 ± 1.4% vs 5.9 ± 0.5%,* p* < 0.001) (Table 1). Anti-hyperglycemic agents were not prescribed in the normoglycemic and prediabetic groups, except for one patient in the prediabetic group who received metformin as a diabetic preventative measure. In the T2DM group, almost all patients (94.5%) received metformin, while 24.7% and 9.6% received sulfonylureas and insulin, respectively (Table 1). Six patients (8.2%) received dipeptidyl-peptidase 4 inhibitors, and only one patient (1.4%) received an alpha-glucosidase inhibitor.

**Table 2:** Univariate logistic regression analysis of osteogenic differentiation impairment across different stages of dysglycemia.

Characteristics	Total (n = 118)	Impaired differentiation (%)	Univariate analysis		
			OR	95% CI	p-value
Normoglycemia	18	2 (11.1)	Ref	–	–
IFG	27	3 (11.1)	1.00	0.15–6.72	1.000
DM ≤ 5 years	30	16 (53.3)	9.14	1.77–47.26	0.008
DM > 5 to ≤ 10 years	24	15 (62.5)	13.33	2.45–72.52	0.003
DM > 10 years	19	17 (89.5)	68.00	8.46–546.65	< 0.0001

Serum pentosidine levels showed no significant difference among the three groups of patients (Table 1). However, the serum pentosidine level in the prediabetic group was the lowest among the three groups. The serum pentosidine level in the group with prediabetes was significantly lower than that in either the group with normoglycemia [2.5 (95% CI 2.0–3.1) ng/mL vs 3.6 (95%CI 2.8–4.6) ng/mL, *p* = 0.029] or the group with T2DM [2.5 (95% CI 2.0–3.1) ng/mL vs 3.4 (95% CI 2.9–4.1) ng/mL, *p* = 0.007). There was a significant difference in serum TNF-α, a proinflammatory marker, between the normoglycemic, prediabetic, and T2DM groups [0.7 (95% CI -0.4–2.2) pg/mL vs 2.2 (95%CI 1.3–3.3) pg/mL vs 2.1 (95% CI 1.6–2.6) pg/mL, *p* = 0.036), suggesting a proinflammatory state in the prediabetic and diabetic groups (Table 1). Serum sRAGE, sRAGE-pentosidine ratios, and IL-β levels were comparable across the three groups.

### Progressive decline in osteogenic differentiation ability during the progression of type 2 diabetes

**Figure 1:**
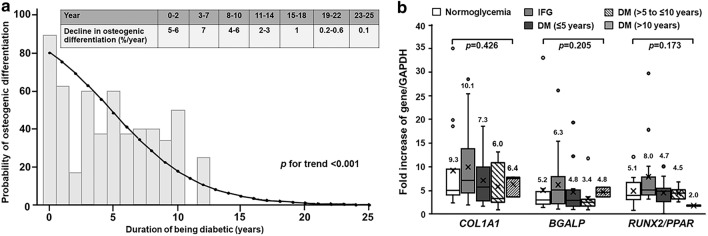
Decline in osteogenic differentiation potential along the progression of type 2 diabetes. (**a**) The probability of osteogenic differentiation impairment along the duration of being diagnosed as diabetic in participants with T2DM. The rate of osteogenic differentiation decreased 4–7% annually during the first 10 years of diagnosis and then decreased at a slower rate. (**b**) The expression of osteogenic differentiation markers in participants who showed a preserved osteogenic differentiation ability**.** Box and whisker plots to show a comparison of the expression of osteoblast-specific genes among five groups of participants, including normoglycemic, prediabetic, diabetic for less than 5 years, diabetic for less than 10 years and diabetic for longer than 10 years. All groups had comparable levels of osteoblast-specific gene expression, including *BGALP* (*p* = 0.426)*, COL1A1* (*p* = 0.205), and *RUNX2/PPARγ* ratio (*RUNX2/PPAR*) (*p* = 0.173).

The PBMC derived from twenty-five participants with T2DM (34.2%) showed expression of all osteoblast-specific marker genes, including *BGLAP, COL1A1*, and the *RUNX2/PPARγ* ratio, indicating differentiation of the cells towards osteoblasts. As shown in Fig. 1a, the probability of differentiation towards osteoblasts of the PBMC progressively declined in line with the longer duration of diabetes. Using univariate logistic regression analysis, the rate of osteogenic differentiation rapidly declined by approximately 4–7% annually for the first 10 years and then slowly declined by approximately 1–3% annually (Fig. 1a). After the 18th year of diabetes, the rate of decline of osteogenic differentiation was lower than 1% annually (Fig. 1a). We next categorized all participants into 5 groups, including normoglycemic, prediabetic, being diabetic for up to 5 years (DM ≤ 5 years), diabetic for up to 10 years (DM > 5 to ≤ 10 years), and diabetic for longer than 10 years (DM > 10 years). Using univariate logistic regression analysis, the rate of osteogenic differentiation impairment in the prediabetic group was comparable to that in the group with normoglycemia (OR 1.00; 95% CI 0.15–6.72, *p* = 1.000) (Table 2). Using the group with normoglycemia as a reference, the rate of osteogenic differentiation impairment significantly increased with the longer duration of diabetes. The rate of osteogenic differentiation impairment progressively increased from 53.3% in the group with diabetes for up to 5 years (OR 9.14; 95% CI 1.77–47.26, *p* = 0.008) to 62.5% in the group with diabetes for up to 10 years (OR 13.33; 95% CI 2.45–72.52, *p* = 0.003) and then to 89.5% in the group with diabetes for longer than 10 years (OR 68.00; 95% CI 8.46–546.65, *p* < 0.001) (Table 2). In PBMC showing differentiation towards osteoblasts, the level of expression of each osteoblast-specific gene, including *BGLAP, COL1A1*, or the *RUNX2/PPARγ* ratio, was comparable among all 5 groups (Fig. 1b).

### Duration of diabetes, in combination with age and estimated glomerular filtration rate, were good predictors of osteogenic differentiation impairment in type 2 diabetes

Forty-eight participants with T2DM (65.8%) showed impairment of osteogenic differentiation as determined by no expression of osteoblast-specific marker genes. The predictive factors for this osteogenic differentiation defect were next to be investigated. Since a higher rate of osteogenic differentiation impairment was shown in the groups with a longer duration of diabetes, the predictive factors for osteogenic differentiation impairment were analyzed by categorizing participants by duration of diabetes (Table 3). By using univariate analysis**,** age, female gender, microvascular complications, sulfonylurea use, insulin use, calcium-channel blocker use, 10-year risk of osteoporotic fractures (FRAX-O) and 10-year risk of hip fractures (FRAX-H) showed a positive trend toward a longer duration of diabetes. In contrast, eGFR, DBP, and the usage of calcium and vitamin D showed a negative trend toward a longer duration of diabetes (Table 3). In terms of biochemical parameters, serum pentosidine showed a negative trend towards a longer duration of diabetes, whereas sRAGE-Pentosidine ratio and IL-1β showed a positive trend.

**Table 3:** Clinical characteristics of the study participants with type 2 diabetes.

Characteristics	Diabetes duration (years)			Test for trend
	≤ 5	> 5 to ≤ 10	> 10	
	(n = 30)	(n = 24)	(n = 19)	*p*-value
Age (years)	56.7 ± 4.1	58.6 ± 7.3	67.4 ± 7.2	< 0.001
Age at diagnosis of diabetes (years)	53.8 ± 4.5	51.3 ± 7.3	52.5 ± 7.6	0.132
Gender (% female)	14 (46.7)	13 (54.2)	15 (79.0)	0.032
BMI (kg/m^2^)	26.3 ± 4.1	25.8 ± 3.0	25.8 ± 5.2	0.459
SBP (mmHg)	133.0 ± 13.8	127.6 ± 16.4	132.9 ± 14.4	0.867
DBP (mmHg)	78.7 ± 9.8	73.8 ± 7.4	72.3 ± 9.5	0.032
DM duration (years)	2.5 (2.0–3.1)	7.2 (6.7–7.8)	14.4 (12.7–16.4)	< 0.001
Microvascular complications (%)	13 (43.3)	12 (50.0)	15 (79.0)	0.020
Macrovascular complication (%)	3 (10.0)	3 (12.5)	4 (21.1)	0.293
FPG (mg/dL)	139.3 (124.1–156.3)	135.2 (123.3–148.2)	130.0 (112.0–150.9)	0.742
HbA1c (%)	7.5 ± 1.6	7.2 ± 0.9	7.2 ± 1.0	0.567
eGFR (mL/min)	85.3 ± 15.1	78.3 ± 19.3	72.4 ± 17.9	0.015
Triglyceride (mg/dL)	113.4 (97.4–135.8)	115.4 (97.3–141.6)	96.3 (84.6–111.7)	0.073
LDL-C* (mg/dL)	88.4 (78.6–99.5)	91.3 (75.8–110.0)	82.0 (69.6–96.6)	0.439
HDL-C# (mg/dL)	51.5 ± 9.5	51.7 ± 12.4	53.2 ± 14.9	0.832
Anti-hyperglycemic agents (%use)				
Metformin	28 (93.3)	23 (95.8)	18 (94.7)	0.801
Sulfonylureas	3 (10.0)	6 (25.0)	9 (47.4)	0.004
Insulin	0	3 (12.5)	4 (21.1)	0.013
Others	3 (10.0)	0	4 (21.1)	0.314
Drugs (% use)				
ACEI or ARB**	19 (63.3)	17 (70.8)	17 (89.5)	0.053
DHP-CCB^##^	13 (43.3)	12 (50.0)	16 (84.2)	0.008
Thiazide-like diuretics	9 (30.0)	5 (20.8)	6 (31.6)	0.996
Statins	25 (83.3)	20 (83.3)	18 (94.7)	0.295
Fibrate	3 (10.0)	1 (4.2)	0	0.128
Calcium and vitamin D	0 (0)	2 (8.3)	3 (15.8)	0.032
FRAX: 10-year risk of hip fractures (FRAX-H) (%)	0.2 (0.2–0.3)	0.4 (0.3–0.6)	1.0 (0.5–2.0)	< 0.001
FRAX:10-year risk of osteoporotic fractures (FRAX-O) (%)	2.0 (1.8–2.2)	2.3 (1.9–2.7)	3.8 (2.9–5.2)	< 0.001
Pentosidine (ng/mL)	4.8 (3.8–6.1)	3.0 (2.3–4.0)	2.4 (1.7–3.4)	0.001
Soluble RAGE (sRAGE) (pg/mL)	450.6 (375.2–541.1)	501.2 (433.5–579.4)	427.6 (341.0–536.3)	0.816
sRAGE-Pentosidine ratio (pg/ng)	93.7 (69.9–125.6)	167.4 (124.9–224.4)	179.5 (116.3–277.1)	0.009
Interleukin-1β (pg/mL)	0.3 (0.2–0.4)	0.3 (0.2–0.5)	0.6 (0.4–0.8)	0.013
Tumor necrosis factor-α (pg/mL)	1.8 (1.1–2.7)	2.4 (1.4–3.6)	2.1 (1.4–3.1)	0.221
Osteogenic differentiation impairment (%)	16 (53.3)	15 (62.5)	17 (89.5)	0.012

*LDL-C: low-density lipoprotein cholesterol; #HDL-C: high-density lipoprotein cholesterol.

**ACEI: angiotensin-converting enzyme inhibitors; **ARB: angiotensin II receptor blockers; ^##^DHP-CCB: dihydropyridine calcium channel blockers.

**Figure 2:**
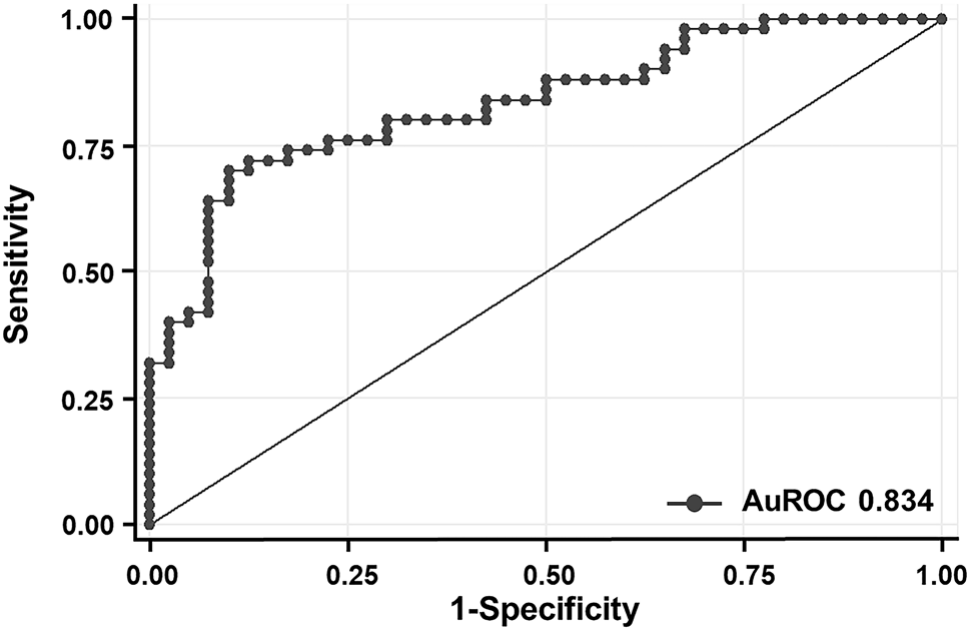
ROC and area under ROC (AuROC) for the prediction of osteogenic differentiation impairment in T2DM. The predictive model that included duration of diabetes, age and eGFR, showed the AuROC of 0.834, indicating a good predictive ability of this model to predict osteogenic differentiation impairment in patients with T2DM.

Multivariate logistic regression analysis was next performed to determine independent risk factors for osteogenic differentiation impairment in T2DM. As shown in Table 4, age, duration of diabetes, and eGFR were risk factors for determining osteogenic differentiation in T2DM. Since age showed a positive trend toward a longer duration of diabetes, multivariate logistic regression analysis with age-adjustment was next performed to determine an independent risk factor for osteogenic differentiation impairment in T2DM. After performing the analysis with age-adjustment, the duration of diabetes and eGFR still significantly influenced osteogenic differentiation in T2DM, indicating both were independent risk factors for osteogenic differentiation in T2DM (Table 4). The longer duration of diabetes and lower eGFR led to the impairment of differentiation towards osteoblasts in T2DM. The AORs of diabetes for up to 5 years (DM ≤ 5 years), diabetes for up to 10 years (DM > 5 to ≤ 10 years), being diabetic for longer than 10 years (DM > 10 years), and eGFR were 15.28 (95% CI 2.35–99.40), 16.91 (95% CI 2.54–112.49), 65.26 (6.64–641.66) and 0.95 (95% CI 0.92–0.99), respectively (Table 4). The AuROC of this predictive model was 0.834 (Fig. 2). For further clinical application of this predictive model, the AOR of each factor was then converted to a ß-coefficient and item score. The assigned score was then generated by rounding the item score to the nearest 0.5 (Table 4). The total score was calculated by combining the assigned scores of each factor as in the following formula: total score = assigned score of DM duration + [age (1)] + [eGFR (− 2.5)]. For example, the total score for a 60-year-old woman who had had diabetes for 8 years and had normal renal function (eGFR of 90 mL/min) was -10, as calculated by [155 + (60 × 1) − (90 × 2.5)]. The cut-off point for the total score of 21.25 was proposed by calculation for the greatest sensitivity and specificity. A total score of at least 21.25 indicated a risk for osteogenic differentiation impairment with sensitivity, specificity, positive predictive value (PPV), and negative predictive value (NPV) of 72%, 87.5%, 87.8%, and 71.4%, respectively. With this predictive model, 78.9% of patients with T2DM were correctly classified for the differentiation ability of their osteoblast progenitors, suggesting good performance of the cut-off level of this predictive model for the prediction of the fate of osteoprogenitor cells in patients with T2DM.

**Table 4:** Multivariate logistic regression analysis with coefficient values of each factor and their assigned score for predicting osteogenic differentiation impairment in type 2 diabetes.

Characteristics	Univariate analysis			Logistic regression			β-coefficient	Item score	Assigned score
	OR	95% CI	*p*-value	AOR*	95% CI	*p*-value			
DM duration (years)									
Normoglycemia	Ref			Ref					
≤ 5 years	9.14	1.77–47.37	0.008	15.28	2.35–99.40	0.004	2.727	149.38	149.5
> 5 to ≤ 10 years	13.33	2.45–72.67	0.003	16.91	2.54–112.49	0.003	2.828	154.92	155
> 10 years	68.00	8.44–548.1	0.000	65.26	6.64–641.66	0.000	4.178	228.90	229
Age (years)	1.09	1.02–1.18	0.018	1.02	0.93–1.12	0.704	0.018	1	1
eGFR (mL/min)	0.96	0.93–0.98	0.003	0.95	0.92–0.99	0.009	− 0.048	− 2.625	− 2.5

## Discussion

The state of low bone turnover with a defect in bone formation was found in T2DM to be partly linked to an alteration of osteoblast development and function found in those patients. Miranda et al.^21^ demonstrated a lower proliferative rate, and a lower expression of RUNX2 and Osterix, transcription factors driving osteoblast differentiation, in osteogenic lineage cells isolated from type 2 diabetic patients who had osteoporotic hip fractures. Sassi et al.^2^ demonstrated a lower number and maturity of circulating osteoblast precursor cells in elderly patients (mean age of 71 ± 6 years) with extremely long-standing T2DM (mean duration of diabetes of 16 ± 2 years). In the younger age group with a shorter duration of diabetes compared to both previously described reports, our previous studies also demonstrated impaired osteogenic differentiation of peripheral blood-derived osteoprogenitor cells isolated from elderly patients (mean age of 63.9 ± 7.2 years) with long-standing T2DM (mean duration of diabetes of 10.9 ± 7.7 years)^22^ and middle-aged patients (mean age of 58.1 ± 6.8 years) with a shorter duration of T2DM (mean duration of DM of 5.5 ± 4.1 years)^23^. Even though multiple previous studies demonstrated a defect in differentiation toward osteoblasts of osteoprogenitor cells in widely varied groups of type 2 diabetic patients, the perspective of the osteogenic differentiation ability of the osteoprogenitor cells has not been established. This study demonstrated the preservation of osteogenic differentiation of the osteoprogenitor cells in normoglycemic and prediabetic individuals while illustrating a progressive decline in the ability of the osteoprogenitor cells to differentiate towards osteoblasts along the natural course of T2DM. The rate of osteogenic differentiation ability rapidly declined by 4–7% annually during the first 10 years of diagnosis and then slowed down to 2–3% and then to less than 1% annually after the 18th year of diagnosis. This study not only showed consistent data involving the impairment of osteogenic differentiation of the osteoprogenitor cells in T2DM but also was the first to show a wider view of osteogenic differentiation ability along the continuum of dysglycemia. Furthermore, our findings may help to explain the mechanism underlying the phenomenon regarding an increase in the prevalence of hip fractures along with the extended duration of diabetes. However, the mechanism leading to a decline in osteogenic differentiation ability along with the progression of diabetes remains to be further elucidated.

Multiple previous in vitro studies reported factors influencing osteoblast development and survival, including hyperglycemia per se^24,25^ and its indirect effects via accelerated AGE accumulation^26–31^ and an increased inflammatory state^19,25,31,32^. Hyperglycemia, AGEs accumulation^38,39^ and a proinflammatory state^32,40,41^ are well-known pathologies of patients with T2DM. However, to date, the direct impact of these three factors on osteoblast development and survival in type 2 diabetic patients remains to be elucidated. In this study, we found that type 2 diabetic patients had significantly higher FPG and HbA1c levels, indicating hyperglycemia, and serum TNF-α, an inflammatory marker, than non-diabetic participants. However, in this study, FPG, HbA1c, serum IL- β, and TNF- α were not found to be independent risk factors for osteogenic differentiation along the progression of T2DM, implying that hyperglycemia and pro-inflammation were not determinants of osteogenic differentiation defects found in our diabetic patients.

In the present study, serum pentosidine, which is a type of AGEs, was not elevated in diabetic patients in comparison to normoglycemic participants and did not increase with the progression of diabetes. As a result, serum pentosidine was not found to be a predictor of osteogenic differentiation defects in our diabetic patients. In contrast to our results, Heidari et al.^42^ demonstrated higher AGEs in diabetic individuals, which were progressively increased with a longer duration of type 2 diabetes. The inconsistency of the two studies may be partly explained by different glycemic control. In comparison to the study of Heidari and colleagues, our enrolled participants had lower HbA1c in all groups of diabetic patients. Furthermore, in our study, the HbA1c did not increased with a longer duration of diabetes (7.5 ± 1.6%, 7.2 ± 0.9%, 7.2 ± 1.0%, *p* = 0.567), whereas in the report by Heidari and colleagues, the HbA1c tended to increase with progression of diabetes (7.5 ± 1.5%, 8.2 ± 2.0%, 8.4 ± 1.9%, 8.2 ± 1.3%, *p* < 0.001). Because hyperglycemia accelerates the accumulation of AGEs, differences in hyperglycemia degree and pattern may result in differences in serum AGE levels along the progression of diabetes between these two studies.

Using multivariate logistic regression analysis, we found three factors that significantly influenced the osteogenic differentiation ability of the circulating osteoprogenitor cells along with the progression of T2DM, including age, duration of diabetes, and eGFR. The older age, the longer duration of diabetes and the lower degree of eGFR led to a defect in osteogenic differentiation of the osteoprogenitor cells. Age and eGFR were shown to have a negative impact on osteoblast development and function. Age was shown to have a negative impact on the osteogenic differentiation ability of mesenchymal stem cells^43–45^. Renal insufficiency, classified by low eGFR, led to an elevation of fibroblast growth factor 23 (FGF23) as early as stage 3 chronic kidney disease^46^. The elevation of FGF23 contributed to the stimulation of Wnt-signaling inhibitors, Sclerostin and Dickoff-1^47^, yielding to the inhibition of the osteoblastic Wnt-signaling pathway which is an essential anabolic pathway for osteoblast development^48^. Duration of diabetes was found to be a novel independent risk factor for osteogenic differentiation impairment of the osteoprogenitor cells in this study, even after adjustment for chronological age. We next produced a predictive model with good diagnostic performance (AuROC 0.834) when these three independent risk factors were included in the model. To facilitate further application in clinical practice, we next determined a cut-off threshold for an increased risk of osteogenic differentiation impairment of the osteoprogenitor cells. According to the calculation for the best sensitivity and specificity, the threshold of 21.25 was proposed as the cut-off point. If the total score of this predictive model was at 21.25 or above, we expected to observe impairment of osteogenic differentiation of osteoprogenitor cells with a sensitivity of 72%, specificity of 87.5%, PPV of 87.8%, NPV of 71.4%, and an accuracy of 78.9%. Therefore, this predictive model with this cut-off level showed good performance for predicting the fate of osteoprogenitor cells in patients with T2DM.

This study had several points of strength in accordance with the originality of our findings. First, this study is the first to illustrate the trajectory of osteoprogenitor cells across a continuum of dysglycemia in T2DM. Second, this study is the first to identify three independent risk factors related to the potential for osteogenic differentiation in patients with T2DM. One factor, the duration of diabetes, has never been reported as an influencing factor for a defect in osteogenic differentiation impairment. All the identified factors are simple factors that could be acquired in routine clinical practice. Third, this study was the first to construct a predictive model to predict a defect in osteogenic differentiation in patients with T2DM. With the proposed cut-off point, this predictive model showed good performance for predicting the fate of osteoprogenitor cells. However, the results should be interpreted with caution due to some limitations. First, because of the limited number of isolated cells in the relatively small 35–40 mL sample of peripheral blood collected from recruited patients, this study lacked evidence on protein expression levels and mineralization studies. This study only demonstrated osteogenic differentiation by the mRNA levels of osteoblast-specific genes. Second, this study was a cross-sectional study, which had several unexpected confounding factors caused by the nature of this type of study. These unexpected confounding factors might influence the results of the study involving risk factors for impairment in osteogenic differentiation. Third, the predictive model proposed in this study was constructed based on the data of diabetic patients in a single center. This may limit the application and transfer of this predictive model to patients with different backgrounds. Therefore, this predictive model should be validated before being applied to widely varying diabetic patients.

In conclusion, this study demonstrated the preservation of osteogenic differentiation of peripheral blood-derived osteoprogenitor cells in normoglycemic and prediabetic individuals but a progressive decline in their ability to differentiate toward osteoblasts along the natural course of T2DM. Therefore, this study provided a perspective on the osteogenic differentiation ability of the osteoprogenitor cells across a continuum of dysglycemia, starting from the normoglycemic condition to long-standing T2DM. In addition, this study demonstrated that age, duration of diabetes, and eGFR were independent risk factors for predicting a defect in differentiation toward osteoblast of the peripheral blood-derived osteoprogenitor cells in T2DM. Using these three independent risk factors, this study was the first to construct a predictive model with good diagnostic performance to predict the fate of peripheral blood-derived osteoprogenitor cells in type 2 diabetic individuals. Since the defect in osteoblast development is linked to a state of low bone turnover and low bone formation in T2DM, which partly leads to impairment in bone quality and an increased risk of fragility fractures in T2DM, this predictive model may be beneficial in clinic for identifying cases at risk for fractures and planning prevention strategies.

## Materials and methods

### Ethics statement

This study was a cross-sectional study, conducted at Maharaj Nakorn Chiang Mai Hospital, Chiang Mai University, Chiang Mai, Thailand. This study was approved by the Research Ethics Committee of the Faculty of Medicine, Chiang Mai University. All participants provided their written informed consent before enrollment in this study. All methods were performed in accordance with the guidelines and regulations of our institute, as well as with the Declaration of Helsinki.

### Study population and sample collection

Individuals with a FPG less than 100 mg/dL were enrolled as a normoglycemic population. Individuals with a FPG of between 100 and 125 mg/dL on at least two occasions, a measurement classified as impaired fasting glucose (IFG) by the American Diabetes Association^49^, were classified as a prediabetic population. Individuals with IFG were excluded if the HbA1c was higher than 6.4%. Individuals with FPG equal or higher than 126 mg/dL on at least two occasions were classified as diabetic^49^. Individuals who were clinical compatible with type 1 diabetes were excluded. The other exclusion criteria were as follows: females with serum creatinine higher than 1.4 mg/dL or males with serum creatinine above 1.5 mg/dL; individuals who use steroids, anti-resorptive agents or anabolic agents for osteoporosis, immunosuppressive agents, thiazolidinedione; and individuals with hematologic or metastatic malignancy.

Venous blood (35–40 mL) was collected from all enrolled participants to isolate the PBMC and determine serum levels of interleukin 1-β (IL1-β) (R&D, Minneapolis, MN, USA), pentosidine (Elabscience Biotechnology, WuHan, Hubei, China), soluble RAGE (sRAGE) (R&D, Minneapolis, MN, USA), and tumor necrosis factor-α (TNF-α) (R&D, Minneapolis, MN, USA) by ELISA. The minimal detectable doses (MDD) of each ELISA are < 1, 0.47, 4.12 and 1.6 pg/mL, respectively. The sensitivity of each ELISA are 1, 0.47, 16.14 and 5.5 pg/ml, respectively. The coefficients of variation (CVs) of intra assay are 4.8, 5.3, 5.7 and 4.7%, respectively. The CVs of inter assay are 5.6, 4.9, 7.7 and 5.8%, respectively. FPG, HbA1c, serum creatinine, high-density lipoprotein cholesterol (HDL-C), low-density lipoprotein cholesterol (LDL-C), and triglyceride levels were assessed using standardized procedures at the central laboratory of the Faculty of Medicine, Chiang Mai University. Glomerular filtration rate (eGFR) was calculated using the Chronic Kidney Disease Epidemiology Collaboration (CKD-EPI) method. Fracture risk estimation was estimated from the Fracture Risk Assessment Tool (FRAX^®^) using the Thailand database^37^.

### Isolation and culture protocol for human peripheral blood-derived mononuclear cells (PBMC)

Peripheral venous blood (35–40 mL) was subjected to density gradient centrifugation and PBMC were isolated and then cultured as described in our previous study^22^. In brief, the plasma was removed from the venous blood by centrifugation at 1500 rpm for 5 min. The remaining cell fraction was first diluted with an equal volume of DMEM (Gibco, Grand Islands, NY, USA) and then overlaid on Histopaque (specific gravity 1.077 g/mL; Sigma-Aldrich, St Louis, MO, USA) and further centrifuged at 4000 rpm for 30 min. The cells in the mononuclear cell layer (PBMC) were plated in 24-well culture plates and cultured in RPMI supplemented with 10% (v/v) fetal bovine serum (Gibco, Grand Islands, NY, USA). The floating cells in the culture wells were removed and the plastic-adhered cells were further cultured in a non-osteogenic-inducing medium, DMEM supplemented with 10% (v/v) fetal bovine serum (Gibco, Grand Islands, NY, USA), for 7–10 days until confluence. To induce osteogenic differentiation, the adhered cells were cultured in a non-osteogenic-inducing medium until they reached 50% confluence. They were then cultured for another 21 days in an osteogenic-inducing medium (DMEM supplemented with 10^–7^ M dexamethasone, 60 μM ascorbic acid, and 10 mM β-glycerophosphate).

### Analysis of the expression of osteoblast-specific genes

The expression of osteoblast-specific genes was determined using real-time PCR as described in our previous studies. In brief, the total RNA (500 ng) was isolated from the cell lysate using the illutraRNA spin Mini Kit (GE Healthcare Life Science, Buckinghamshire/Little Chalfont, UK) following the manufacturer’s instructions. The isolated total RNA of each sample was reverse transcribed into cDNA using an iScript™cDNA Synthesis Kit (Bio-Rad, Hercules, CA, USA) following the manufacturer’s protocol. The cDNA was subsequently analyzed by reversed transcription PCR (RT-PCR) using Sso7d fusion enzyme technology according to the manufacturer’s instructions (Bio-Rad, Hercules, CA, USA). The PCR protocol consisted of 45 cycles of 5 s at 95 °C, 10 s at 60 °C, and 30 s at 72 °C using the Applied Biosystems 7500/7500 Fast Real-Time PCR system. Total RNA was isolated from both non-osteogenic and osteogenic-inducing cells and used to analyze osteoblast-specific genes, including *BGLAP*, *COL1A1*, and *RUNX2* for the representation of osteoblast differentiation and *PPARγ* for the representation of signaling against osteoblast differentiation. Multiple osteoblast-specific genes, including *BGLAP*, *COL1A1*, and *RUNX2*, were expressed during the process of osteoblast differentiation^33,50^ and were persistently elevated in human osteoblasts^50,51^. Therefore, the osteogenic differentiation, in this study, was defined by the increment of expression of all osteoblast-specific marker genes, including *BGLAP* and *COL1A1*, as well as the increment of the *RUNX2/PPARγ* ratio. The *GAPDH* expression was used for normalization of the relative expression levels for each primer set by the 2^(-ΔΔCT)^ method. The primers used for real-time PCR were as follows: for *BGLAP*, forward 5′-GAAGCCCAGCGGTGCA-3′ and reverse 5′-CACTACCTCGCTGCCCTCC-3′; for *COL1A1*, forward 5′-CAGCCGCTTCACCTA CAGC-3′ and reverse 5′-TTTTGTATTCAATCACTGTCTTGCC-3′; for *PPARγ*, forward 5′-AAAGAAGCCAACACTAAACC-3′ and reverse 5′-CTTCCATTACGGAGAGATCC-3′; for *RUNX2*, forward 5′-TCTTAGAACAAATTCTGCCCTTT-3′ and reverse 5′-TGCTTTGGTCTTGA AATCACA-3′; for *GAPDH*, forward 5′-CCCTTCATTGACCTCAACTA-3′ and reverse 5′-AGATGATGACCCTTTTGGCT-3′. All primers were purchased from Invitrogen.

### Statistical analysis

Statistical analysis was conducted using SPSS version 23.0. All normally distributed continuous variables were reported as mean ± standard deviation (mean ± SD), whereas non-normally distributed continuous variables were transformed and then reported as mean and 95% confidence interval (CI). All categorical variables were presented as counts and percentages. An independent t-test was used for univariable comparative statistics for two sets of continuous data. An ANOVA test was used for univariable comparative statistics for at least three sets of normally distributed continuous data, while the Kruskal–Wallis test was used to compare at least three sets of continuous data that did not show a normal distribution. A chi-square test was used for univariable comparative statistics for all categorical variables, the exception being any categorical variable with small counts, which was analyzed using Fisher’s exact test. A univariate logistic regression analysis was performed to determine the rate of osteogenic differentiation impairment along with the progression of diabetes. Multivariate logistic regression analysis was performed to determine predictive factors for osteogenic differentiation impairment at different stages of type 2 diabetes and was used to develop the predictive model for the defects in osteogenic differentiation. Univariable and multivariable regression analyses were conducted by logistic regression analysis and the results were reported as odds ratio (OR) and adjusted odds ratio (AOR) and 95% confidence interval (CI), respectively. The final predictive model was developed by stepwise backward selection by removing the factors with a *p*-value > 0.1. To create an item score, the AOR was converted to the ß-coefficient. The ß-coefficient of each factor was divided by the smallest ß-coefficient in the final model and rounded to the nearest 0.5. The total score was calculated by combining all the item scores. The cut-off point for the total score was calculated for the greatest sensitivity and specificity. The areas under the receiver operating characteristics curves (AuROC) were plotted to determine the diagnostic performance of that identified predictive model. Sensitivity, specificity, positive predictive value (PPV), negative predictive value (NPV), and accuracy were also calculated for the predictive model. A *p*-value of less than 0.05 was used as a measure of statistical significance. A sample size calculation was performed to estimate the number needed to show the non-inferiority of osteogenic differentiation in the normoglycemic group compared to the prediabetic group, as well as the difference in osteogenic differentiation in the normoglycemic group compared to each of the diabetic groups. A sample size of at least 16 participants in the normoglycemic group and 23 patients in the prediabetic group was estimated to give 80% power at the 5% significance level to detect a non-inferiority of osteogenic differentiation in the prediabetic group compared to the normoglycemic group at a 20% margin of equivalence. A sample size of at least 14 participants in each group of diabetics was estimated to give 80% power at the 5% significance level to detect differences in osteogenic differentiation between the normoglycemic group and each of the diabetic groups.

## Data Availability

All data generated or analyzed during this study are included in this published article.

## References

[1] Shu A (2012). Bone structure and turnover in type 2 diabetes mellitus. Osteoporos Int..

[2] Sassi F (2018). Type 2 diabetes affects bone cells precursors and bone turnover. BMC Endocr. Disord..

[3] Paccou J (2016). Bone microarchitecture in men and women with diabetes: The importance of cortical porosity. Calcif. Tissue Int..

[4] Vestergaard P (2007). Discrepancies in bone mineral density and fracture risk in patients with type 1 and type 2 diabetes—A meta-analysis. Osteoporos Int..

[5] Yamamoto M, Yamaguchi T, Yamauchi M, Kaji H, Sugimoto T (2007). Bone mineral density is not sensitive enough to assess the risk of vertebral fractures in type 2 diabetic women. Calcif. Tissue Int..

[6] Ma L (2012). Association between bone mineral density and type 2 diabetes mellitus: A meta-analysis of observational studies. Eur. J. Epidemiol..

[7] Oei L (2013). High bone mineral density and fracture risk in type 2 diabetes as skeletal complications of inadequate glucose control: The Rotterdam Study. Diabetes Care.

[8] Schneider AL (2013). Diabetes and risk of fracture-related hospitalization: The Atherosclerosis Risk in Communities Study. Diabetes Care.

[9] Li CI (2015). Glycated hemoglobin level and risk of hip fracture in older people with type 2 diabetes: A competing risk analysis of Taiwan diabetes cohort study. J. Bone Miner. Res..

[10] Park HY, Han K, Kim Y, Kim YH, Sur YJ (2021). The risk of hip fractures in individuals over 50 years old with prediabetes and type 2 diabetes—A longitudinal nationwide population-based study. Bone.

[11] Jiajue R (2014). Suppressed bone turnover was associated with increased osteoporotic fracture risks in non-obese postmenopausal Chinese women with type 2 diabetes mellitus. Osteoporos Int..

[12] Kubihal S, Gupta Y, Goyal A, Kalaivani M, Tandon N (2022). Bone microarchitecture, bone mineral density and bone turnover in association with glycemia and insulin action in women with prior gestational diabetes. Clin. Endocrinol. (Oxf.).

[13] Iki M (2019). Hyperglycemic status is associated with an elevated risk of osteoporotic fracture in community-dwelling elderly Japanese men: The Fujiwara-kyo osteoporosis risk in men (FORMEN) cohort study. Bone.

[14] Chen C (2020). Trends in bone mineral density, osteoporosis, and osteopenia among US adults with prediabetes, 2005–2014. Diabetes Care.

[15] Dominic E (2020). Metabolic factors and hip fracture risk in a large Austrian cohort study. Bone Rep..

[16] Leite Duarte ME, da Silva RD (1996). Histomorphometric analysis of the bone tissue in patients with non-insulin-dependent diabetes (DMNID). Rev. Hosp. Clin. Fac. Med. Sao Paulo.

[17] Pramojanee SN, Phimphilai M, Kumphune S, Chattipakorn N, Chattipakorn SC (2013). Decreased jaw bone density and osteoblastic insulin signaling in a model of obesity. J. Dent. Res..

[18] Ross DS (2021). Distinct effects of a high fat diet on bone in skeletally mature and developing male C57BL/6J mice. Nutrients.

[19] Colombo JS (2011). Delayed osteoblast differentiation and altered inflammatory response around implants placed in incisor sockets of type 2 diabetic rats. Clin. Oral. Implants Res..

[20] Hamann C (2011). Delayed bone regeneration and low bone mass in a rat model of insulin-resistant type 2 diabetes mellitus is due to impaired osteoblast function. Am. J. Physiol. Endocrinol. Metab..

[21] Miranda C (2016). Influence of high glucose and advanced glycation end-products (ages) levels in human osteoblast-like cells gene expression. BMC Musculoskelet. Disord..

[22] Phimphilai M, Pothacharoen P, Kongtawelert P, Chattipakorn N (2017). Impaired osteogenic differentiation and enhanced cellular receptor of advanced glycation end products sensitivity in patients with type 2 diabetes. J. Bone Miner. Metab..

[23] Phimphilai M, Pothacharoen P, Kongtawelert P (2021). Age-influenced receptors of advanced glycation end product overexpression associated with osteogenic differentiation impairment in patients with type 2 diabetes. Front. Endocrinol..

[24] Liu C, Jiang D (2017). High glucose-induced LIF suppresses osteoblast differentiation via regulating STAT3/SOCS3 signaling. Cytokine.

[25] Yang L, Liu J, Shan Q, Geng G, Shao P (2020). High glucose inhibits proliferation and differentiation of osteoblast in alveolar bone by inducing pyroptosis. Biochem. Biophys. Res. Commun..

[26] Alikhani M (2007). Advanced glycation end products stimulate osteoblast apoptosis via the MAP kinase and cytosolic apoptotic pathways. Bone.

[27] Franke S, Siggelkow H, Wolf G, Hein G (2007). Advanced glycation endproducts influence the mRNA expression of RAGE, RANKL and various osteoblastic genes in human osteoblasts. Arch. Physiol. Biochem..

[28] Sanguineti R, Storace D, Monacelli F, Federici A, Odetti P (2008). Pentosidine effects on human osteoblasts in vitro. Ann. N. Y. Acad. Sci..

[29] Okazaki K (2012). Advanced glycation end products (AGEs), but not high glucose, inhibit the osteoblastic differentiation of mouse stromal ST2 cells through the suppression of osterix expression, and inhibit cell growth and increasing cell apoptosis. Calcif. Tissue Int..

[30] Notsu M (2014). Advanced glycation end product 3 (AGE3) suppresses the mineralization of mouse stromal ST2 cells and human mesenchymal stem cells by increasing TGF-β expression and secretion. Endocrinology.

[31] Liu J (2016). AGEs induce apoptosis in rat osteoblast cells by activating the caspase-3 signaling pathway under a high-glucose environment in vitro. Appl. Biochem. Biotechnol..

[32] Liu C (2016). Adiponectin, TNF-α and inflammatory cytokines and risk of type 2 diabetes: A systematic review and meta-analysis. Cytokine.

[33] Valenti MT (2008). Gene expression analysis in osteoblastic differentiation from peripheral blood mesenchymal stem cells. Bone.

[34] Cesselli D (2009). Multipotent progenitor cells are present in human peripheral blood. Circ. Res..

[35] Yang HS (2011). Enhancement of human peripheral blood mononuclear cell transplantation-mediated bone formation. Cell Transplant..

[36] Phimphilai M, Pothacharoen P, Chattipakorn N, Kongtawelert P (2022). Receptors of advanced glycation end product (RAGE) suppression associated with a preserved osteogenic differentiation in patients with prediabetes. Front. Endocrinol. (Lausanne).

[37] Centre for Metabolic Bone Diseases, University of Sheffield. *The Fracture Risk Assessment Tool (FRAX®).* https://www.sheffield.ac.uk/FRAX/tool.aspx?country=57 (2008).

[38] Vlassara H, Striker GE (2013). Advanced glycation endproducts in diabetes and diabetic complications. Endocrinol. Metab. Clin. N. Am..

[39] Asadipooya K, Uy EM (2019). Advanced glycation end products (AGEs), receptor for ages, diabetes, and bone: Review of the literature. J. Endocr. Soc..

[40] Wang X (2013). Inflammatory markers and risk of type 2 diabetes: A systematic review and meta-analysis. Diabetes Care.

[41] Luc K, Schramm-Luc A, Guzik TJ, Mikolajczyk TP (2019). Oxidative stress and inflammatory markers in prediabetes and diabetes. J. Physiol. Pharmacol..

[42] Heidari F (2020). Advanced glycation end-products and advanced oxidation protein products levels are correlates of duration of type 2 diabetes. Life Sci..

[43] Stenderup K, Justesen J, Clausen C, Kassem M (2003). Aging is associated with decreased maximal life span and accelerated senescence of bone marrow stromal cells. Bone.

[44] Moerman EJ, Teng K, Lipschitz DA, Lecka-Czernik B (2004). Aging activates adipogenic and suppresses osteogenic programs in mesenchymal marrow stroma/stem cells: the role of PPAR-gamma2 transcription factor and TGF-beta/BMP signaling pathways. Aging Cell.

[45] Infante A, Rodríguez CI (2018). Osteogenesis and aging: Lessons from mesenchymal stem cells. Stem Cell Res. Ther..

[46] Pavik I (2013). Secreted Klotho and FGF23 in chronic kidney disease Stage 1 to 5: A sequence suggested from a cross-sectional study. Nephrol. Dial. Transplant..

[47] Carrillo-López N (2016). Direct inhibition of osteoblastic Wnt pathway by fibroblast growth factor 23 contributes to bone loss in chronic kidney disease. Kidney Int..

[48] Baron R, Kneissel M (2013). WNT signaling in bone homeostasis and disease: From human mutations to treatments. Nat. Med..

[49] American Diabetes Association (2022). 2. Classification and diagnosis of diabetes: Standards of medical care in diabetes-2022. Diabetes Care.

[50] Lian JB, Stein GS (1995). Development of the osteoblast phenotype: Molecular mechanisms mediating osteoblast growth and differentiation. Iowa Orthop. J..

[51] Czekanska EM, Stoddart MJ, Ralphs JR, Richards RG, Hayes JS (2014). A phenotypic comparison of osteoblast cell lines versus human primary osteoblasts for biomaterials testing. J. Biomed. Mater. Res. A.

